# Mapping the physiological landscape of body movements during nocturnal sleep and wakefulness and their cardiovascular correlates with a wearable multi-sensor array

**DOI:** 10.1038/s41598-025-29723-7

**Published:** 2025-11-29

**Authors:** Marcello Sicbaldi, Paola Di Florio, Luca Palmerini, Raffaele Ferri, Lorenzo Chiari, Alessandro Silvani

**Affiliations:** 1 Department of Electrical, Electronic, and Information Engineering “Guglielmo Marconi”, University of Bologna, Bologna, Italy; 2 Department of Biomedical and Neuromotor Sciences, University of Bologna, Piazza di Porta San Donato 2, Bologna, 40126 Italy; 3 Health Sciences and Technologies-Interdepartmental Center for Industrial Research, University of Bologna, Bologna, Italy; 4 Oasi Research Institute-IRCCS, Troina, Italy

**Keywords:** Wearable sensors, Sleep, Movements, Heart rate, Pulse wave amplitude, Physiology., Biomarkers, Cardiology, Engineering, Neuroscience, Physiology

## Abstract

Spontaneous motor activity is a physiological feature of sleep and is enhanced in sleep-related movement disorders. We aimed to develop a measurement and analysis approach to movements during nocturnal sleep and wakefulness, as well as their cardiovascular correlates, entirely based on a wearable multi-sensor array, and to test its feasibility and internal consistency. Twelve healthy participants slept overnight at home wearing an array of seven wearable sensors: five accelerometers, a photoplethysmograph, and an electrocardiograph. Sleep-wake states were determined from wrist actigraphy and sleep diaries. We developed an algorithm for detecting movements based on body segment acceleration and validated it against visual annotations of accelerometer tracings. The F1 scores ranged from 90.6% to 94.4% for different segments. Our algorithm indicated that movements during sleep were significantly fewer than during wakefulness (21.4 ± 1.5 vs. 90.4 ± 6.2 per hour, *p* < 0.001), with relatively more segmental (42.8% vs. 15.0%) and lower-body (15.3% vs. 5.7%) movements and relatively fewer global movements (23.7% vs. 66.1%). Heart rate started to increase and pulse wave amplitude started to decrease at, or up to 6 s before, movement onset, depending on movement type and wake-sleep state, consistent with cardiac activation and peripheral vasoconstriction due to central autonomic commands. The magnitude of these cardiovascular responses positively correlated with intensity particularly of global movements (*p* < 0.001). These results demonstrate the feasibility and internal consistency of a new approach, entirely based on wearables sensors, to map the physiological landscape of body movements and their cardiovascular correlates during nocturnal sleep and wakefulness.

**Supplementary Information:**

The online version contains supplementary material available at 10.1038/s41598-025-29723-7.

## Introduction

Spontaneous motor activity is a distinct feature of physiological sleep^1^ and is enhanced in sleep-related movement disorders^2–5^. Video-polysomnography (PSG), which includes multiple electroencephalographic and electromyographic leads and heart rate (HR) and respiratory sensors, along with video recordings, is central to the diagnosis and characterization of sleep-related motor activity. Electromyographic recordings are necessary to score non-periodic and periodic leg movements during sleep (LMS)^6^, which are an important clinical feature supporting the diagnosis of restless legs syndrome^7^, as well as large muscle group movements during sleep (LMMS)^8^. Arousals from non-rapid-eye-movement (non-REM) sleep^9^ and LMS^10–12^ are preceded and accompanied by increases in HR and followed by increases in arterial blood pressure, which, in turn, may be followed by decreases in HR below baseline^10,13,14^. This pattern of cardiovascular changes is consistent with movement-related central autonomic commands on the heart and blood vessels completely or partially overriding the arterial baroreceptor reflex control of HR^15^. Similarly, graded central autonomic commands play a key role in increasing HR and arterial blood pressure during movements in wakefulness^16^. The HR increases associated with periodic leg movements in sleep (PLMS) are enhanced in restless legs syndrome^14,17^, reduced in narcolepsy type 1^18^, and severely reduced in multiple system atrophy^19^, suggesting that they also carry relevant information on sleep disorder pathophysiology.

While monitoring spontaneous motor activity during sleep and its cardiovascular correlates may yield valuable insights into the occurrence and severity of sleep-wake disorders and their response to treatment, the complexity, logistics, and cost of video-PSG make it unrealistic to scale to long periods and at the population level. Sleep actigraphy, based on a wrist accelerometer, has low specificity but high sensitivity for detecting sleep^20^, and it is actively explored for applications in diagnosing, monitoring, and managing various sleep disorders^21^. The combination of wrist actigraphy with inertial sensors on different body segments may provide comprehensive information on movements during nocturnal sleep and wakefulness. Further combination of these sensors with wearable sensors recording HR and pulse wave amplitude (PWA) may inform on changes in sympathetic and parasympathetic activity to the heart^22^and in sympathetic peripheral vasoconstriction^23^, respectively.

For these reasons, we aimed to develop a measurement and analysis approach to movements during nocturnal sleep and wakefulness, as well as their HR and PWA correlates, based on a wearable multi-sensor array and to test its feasibility and internal consistency. We developed a procedure to automatically detect movements of different body segments (wrists, ankles, lower back) based on their bursts of acceleration and validated it against visual inspection of acceleration time series by two independent investigators. We then applied our procedure to characterize the landscape of body movements during nocturnal sleep and wakefulness in a sample of young healthy subjects. Finally, we extensively characterized the HR and PWA correlates of the body movements depending on their location, extent, and relationship to actigraphy-defined nocturnal sleep and wakefulness.

## Results

### Optimization and validation of the thresholds for movement detection

The inter-rater agreement for movement detection by visual assessment of acceleration time series was 87.4% (1079 agreements, 156 disagreements). The sensitivity, precision, and F1-score for each movement detection threshold are reported in Supplementary Fig. 1. The selected thresholds were 20 mg for the wrists and 15 mg for the ankles and the trunk. The values of sensitivity, precision, and F1 score ranged from 93.5% to 94.7%, from 88.8% to 95.1%, and from 90.6% to 94.4%, respectively.

### Movement topography during nocturnal sleep and wakefulness

The durations of diary-defined sleep period time (dSPT), actigraphy-defined sleep (aS), and actigraphy-defined wakefulness (aW) were 451.9 ± 10.0 min, 401.2 ± 11.8 min, and 45.6 ± 6.7 min, respectively. The efficiency of aS was 88.8 ± 1.8%. The number of awakenings, as determined by wrist actigraphy, was 2.5 ± 0.2 per hour of aS.

A total of 2466 movements were detected during dSPT, with 1698 occurring during aS and 768 during aW. As expected, and as shown in Table 1, the total movement index during dSPT was significantly higher than during aS and lower than during aW. Similarly, the average movement duration and magnitude during dSPT were both significantly higher than during aS and significantly lower than during aW.

**Table 1:** Movement metrics during the night.

Movement Metric	aS	dSPT	aW	Statistical Comparison
Index (/h)	21.4 ± 1.5	27.7 ± 2.1	90.4 ± 6.2	aW > dSPT > aS (*p* < 0.001, t-test)
Duration (s)	4.5 ± 0.4	7.7 ± 0.6	16.9 ± 2.0	dSPT > aS (*p* < 0.001, Wilcoxon test) dSPT < aW (*p* < 0.001, t-test)
Magnitude (mg)	377 ± 63	981 ± 119	2506 ± 240	dSPT > aS (*p* < 0.001, Wilcoxon test) dSPT < aW (*p* < 0.001, t-test)

aS and aW: actigraphy-defined sleep and wakefulness, respectively. dSPT = diary-defined sleep period time.

**Fig. 1:**
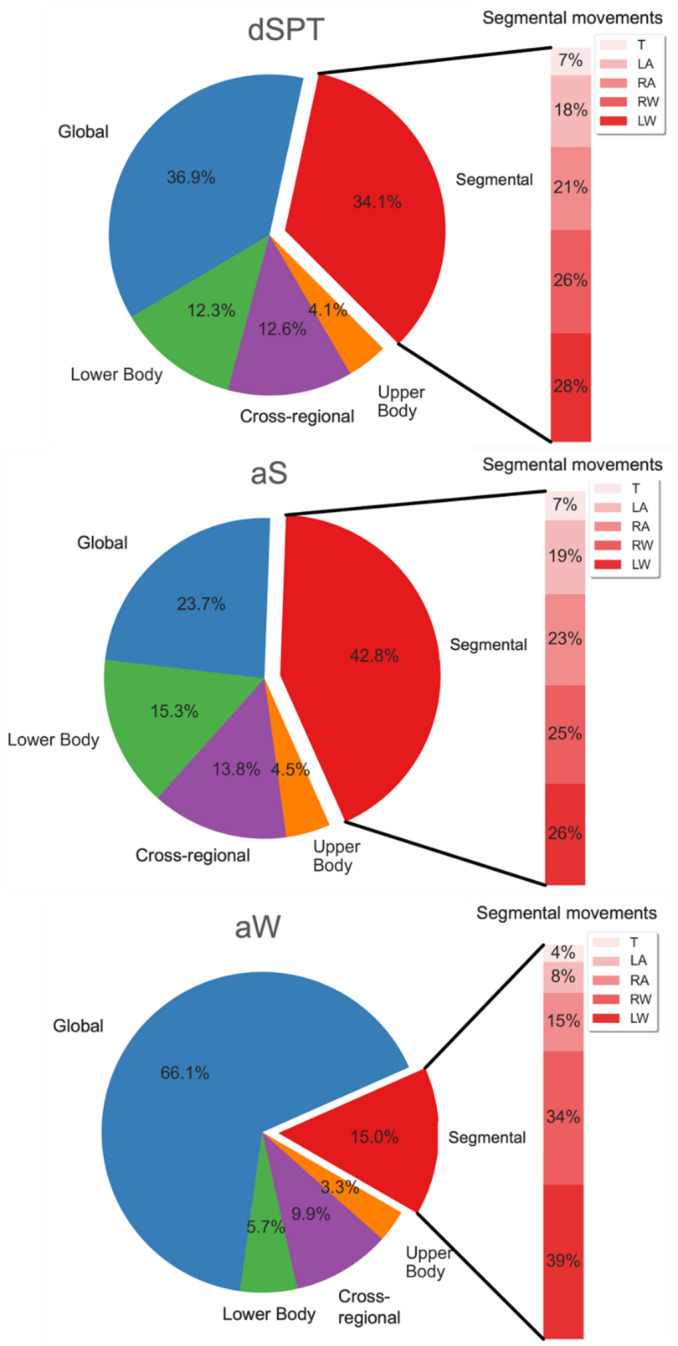
Movement topography during the diary-defined sleep period time (dSPT, top) and actigraphy-defined sleep (aS, middle) and wakefulness (aW, bottom). T = trunk, LA = non-dominant ankle, RA = dominant ankle, RW = dominant wrist, LW = non-dominant wrist.

Movements were classified as segmental (or localized) when confined to a single body segment^1^, and as multisegmental when involving two or more segments. Multisegmental movements were further categorized into global and regional movements. Global movements involved both wrists, both ankles, and the lower back, indicative of whole-body activation. Regional movements included upper-body movements, involving both wrists or one wrist and the lower back, lower-body movements, involving both ankles or one ankle and the lower back, and cross-regional movements, comprising any other combination of non-contiguous segments.

During dSPT, most movements were classified as either global or segmental. Fewer movements involved only the lower-body, only the upper-body, or were cross-regional in type. Segmental and lower-body movements were a significantly larger fraction of total movements during aS than during aW (*p* < 0.001 and *p* < 0.005, Wilcoxon signed-rank test and paired t-test, respectively). In contrast, global movements were a significantly larger fraction of total movements during aW than during aS (*p* < 0.001, paired t-test). Postural changes during aS were rare, with a mean index of 3.2 per hour of aS. During aW, they occurred significantly more often than during aS (*p* < 0.001, paired t -test), accounting for 34.3 ± 5.2% of global movements, with a mean index of 12.0/h. The overall topographical distribution of movements during dSPT, aS, and aW is shown in Fig. 1.

The movement index during the second half of the dSPT was significantly higher than during the first half for global (*p* < 0.01, Wilcoxon test), lower-body, and cross-regional movements (both *p* < 0.01, paired t-test). A similar pattern was observed for movements during aS, but not during aW. For segmental movements, the difference between the two halves of dSPT, either overall or in aW, did not reach statistical significance, and no difference was observed for upper-body movements (Supplementary Fig. 2). The absolute values for the movement index, duration, and magnitude across movement categories during dSPT, aS, and aW are reported in Supplementary Table 1.

### Cardiovascular correlates of movements

Based on the artifact detection and correction procedures described in the Methods, 1.5% of all heartbeats were discarded and 0.1% were interpolated before analysis; for PWA, 4.2% of all heartbeats were discarded and 0.8% interpolated.

### Segmental movements

**Fig. 2:**
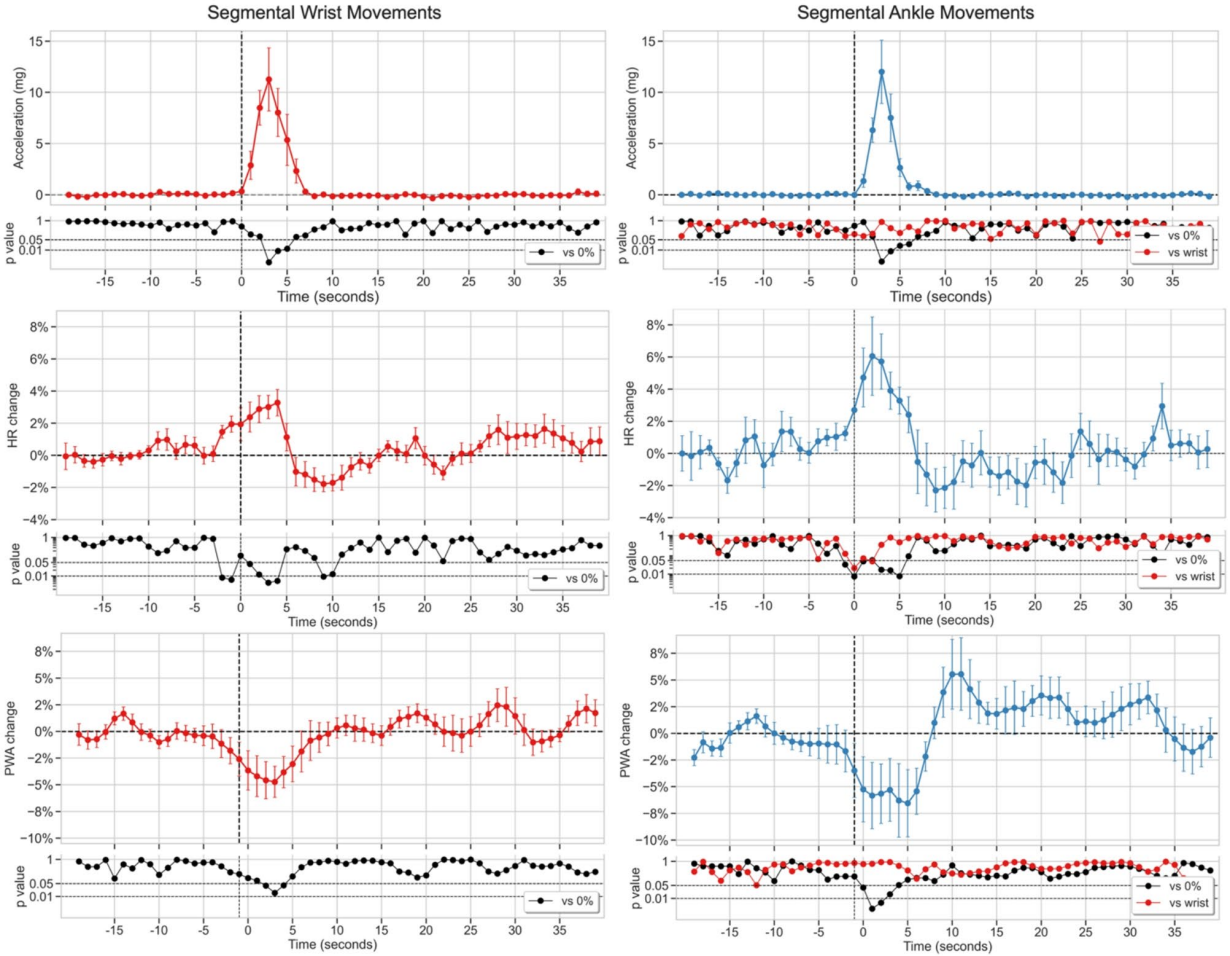
Grand-averaged time-courses of changes in acceleration (peak-to-peak amplitude of the acceleration signal vector magnitude, top row), heart rate (HR, middle row), and pulse wave amplitude (PWA, bottom row) associated with segmental wrist (left column) and ankle (right column) movements during the diary-defined sleep period. Data are presented as mean ± SEM of percent change from baseline with *N* = 9 subjects. Time zero (T = 0) indicates movement onset. P-values are shown on a logarithmic scale.

Due to the markedly lower number of segmental movements meeting the analysis criteria (including absence of HR and photoplethysmography (PPG) artifacts longer than 10 s and no other movements within 30 s before or after the target movement) during aW compared to aS (14 vs. 252), the cardiovascular correlates of segmental movements during dSPT closely reflected those observed during aS. Segmental movements involving the trunk were particularly rare during dSPT (*n* = 9), compared to those involving the ankles (*n* = 79) or wrists (*n* = 158). Therefore, the analysis focused on segmental wrist and ankle movements during dSPT (Fig. 2).

These movements were associated with biphasic HR responses, with early HR increases (5.4 ± 1.0% at 3 s post-onset) beginning 1-2 s before movement onset, which were significantly greater for ankle movements than for wrist movements, followed by later HR decreases (−2.1 ± 0.7%), which reached significance for wrist movements. PWA decreased during the movements (−5.8 ± 2.6%), with a non-significant trend toward increases after ankle movements..

No significant correlation was observed between segmental movement magnitude and either the HR peak or the PWA trough (Supplementary Fig. 3), suggesting that cardiovascular responses were relatively stereotyped, regardless of variability in movement magnitude. In constrast, a significant negative correlation was found between HR peak and PWA trough during segmental movements, both at the wrist (*r* = −0.31, *p* < 0.001) and the ankle (*r* = −0.54, *p* < 0.001). This finding indicates that greater sympathetic activation and/or parasympathetic withdrawal at the cardiac level was associated with more pronounced peripheral vasoconstriction, consistent with increased sympathetic tone also at the vascular level.

### Regional movements

**Fig. 3:**
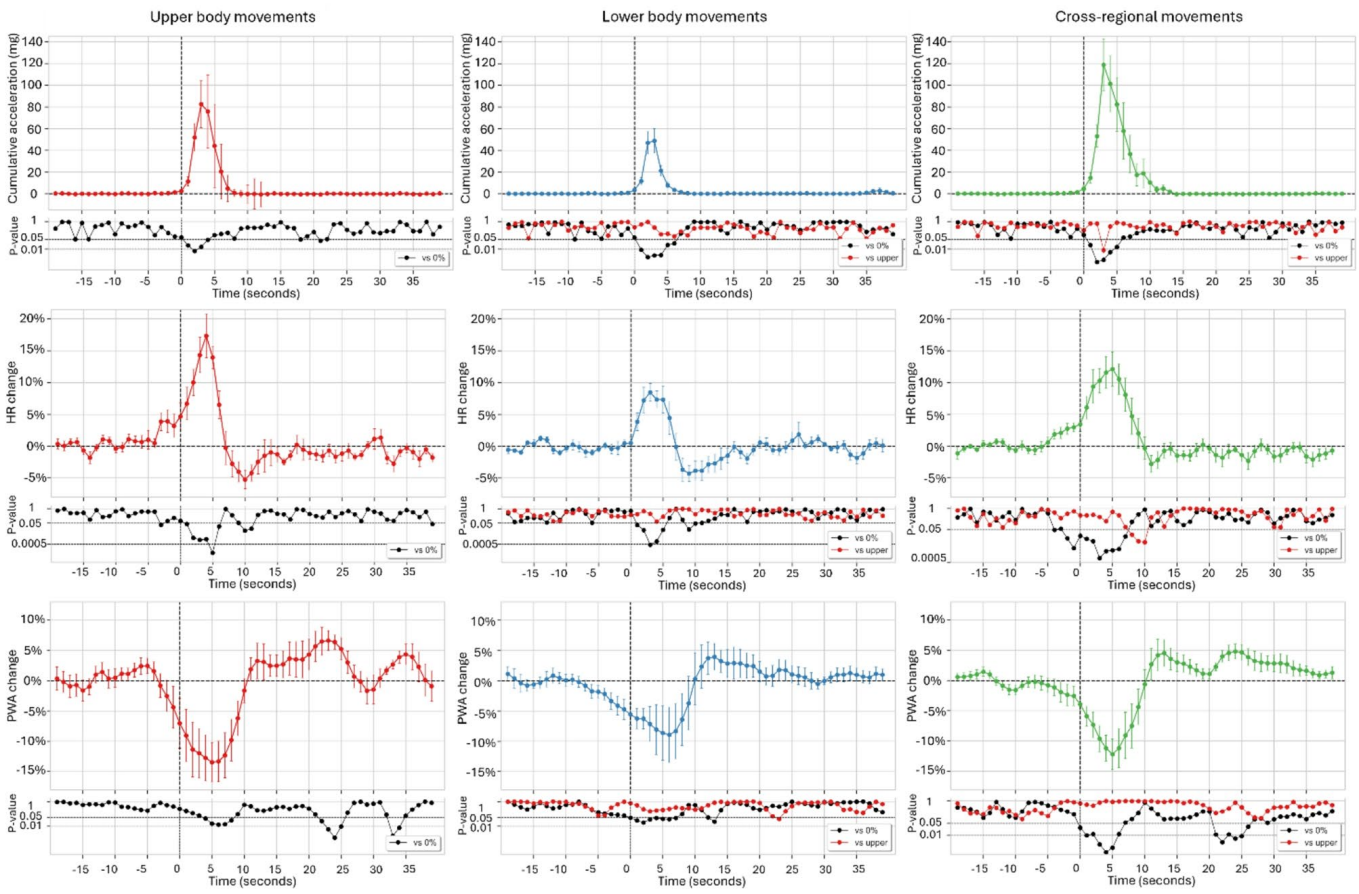
Grand-averaged time courses of changes in acceleration (peak-to-peak amplitude of the acceleration signal vector magnitude, top row), heart rate (HR, middle row), and pulse wave amplitude (PWA, bottom row) associated with regional movements during the diary-defined sleep period. Data are presented as mean ± SEM of percent change from baseline with *N* = 9 subjects. Time zero (T = 0) indicates movement onset. P-values are displayed on a logarithmic scale.

Due to the substantially lower number of lower-body (6 vs. 90), upper-body (7 vs. 40), and cross-regional (16 vs. 84) movements meeting the inclusion criteria for this analysis during aW compared to aS, the cardiovascular correlates of regional movements during dSPT closely reflected those observed during aS. Consequently, the analysis focused on lower-body, upper-body, and cross-regional movements occurring during dSPT (Fig. 3).

Both lower-body and upper-body movements were associated with biphasic HR changes: an initial HR increase, which began at movement onset for lower-body movements (8.5 ± 1.4%) and 3 s before onset for upper-body movements (17.4 ± 3.4%), followed by a delayed HR decrease. Cross-regional movements, by contrast, elicited monophasic HR increases (12.1 ± 2.7%) starting approximately 4 s before movement onset, without a subsequent decrease. PWA responses exhibited biphasic patterns opposite to those of HR, with significant reductions at movement onset followed by variable later increases, with peaks occurring between 10 s and 35 s after the movement, depending on the movement type.

Positive correlations were found between regional movement magnitude and HR peak (Supplementary Fig. 4), reaching significance for lower-body movements (*r* = 0.24, *p* < 0.001). This suggests that larger lower-body movements are associated with greater sympathetic activation and/or parasympathetic withdrawal at the cardiac level. In addition, negative correlations were observed between movement magnitude and PWA trough, reaching significance for cross-regional movements (*r* = −0.46, *p* < 0.001), indicating a link between increased movement magnitude and enhanced sympathetic vasoconstriction. Finally, HR peak and PWA trough were significantly negatively correlated for lower-body (*r* = −0.60, *p* < 0.001) and cross-regional movements (*r* = −0.56, *p* < 0.001), suggesting that stronger cardiac autonomic responses were accompanied by more pronounced peripheral vasoconstriction.

### Global movements

**Fig. 4:**
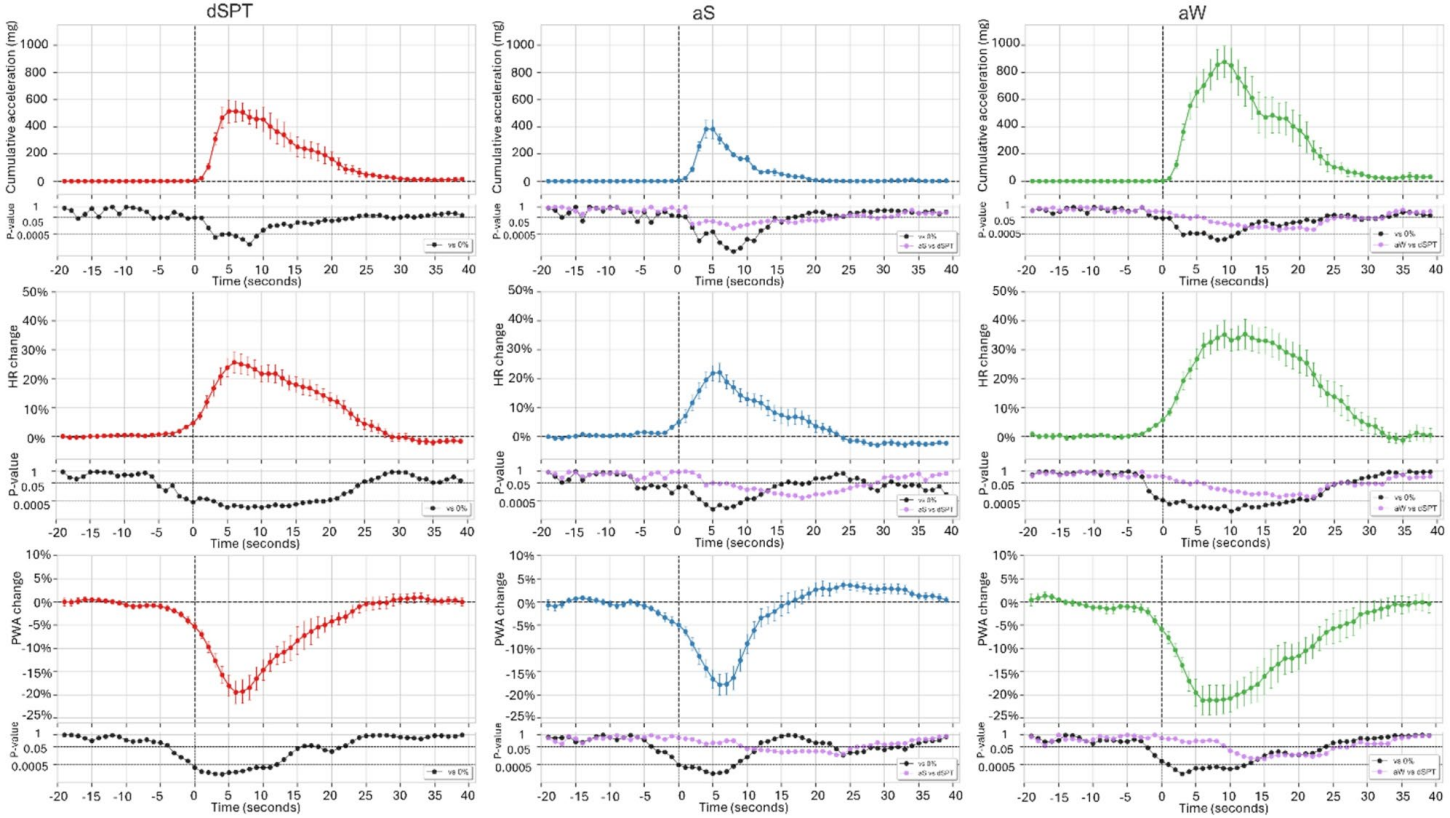
Grand-averaged time-courses of changes in acceleration (peak-to-peak amplitude of the acceleration signal vector magnitude, top row), heart rate (HR, middle row), and pulse wave amplitude (PWA, bottom row) associated with global movements during the diary-defined sleep period time (dSPT, left column) and actigraphy-defined sleep (aS, middle column) and wakefulness (aW, right column). Data are presented as mean ± SEM of percent changes from baseline with *N* = 9 subjects. Time zero (T = 0) indicates movement onset. P-values are shown on a logarithmic scale.

Since the number of global movements meeting the analysis criteria was comparable during aW and aS (403 vs. 508), the cardiovascular correlates of global movements during aS did not approximate those observed during dSPT. Therefore, results are hereafter reported separately for dSPT, aS, and aW (Fig. 4).

Global movements were associated with monophasic HR increases (25.6 ± 3.7%), beginning 2–6 s before movement onset, followed by a significant late HR decrease only during aS (−3.0 ± 0.8%, starting 28 s after movement onset). Similarly, these movements were accompanied by monophasic decreases in PWA (−19.4 ± 2.5%), starting 2–3 s before movement onset, with a significant late increase observed only during aS (3.7 ± 0.9%, starting 23 s after movement onset). Compared to dSPT, global movements during aS were smaller and characterized by smaller and/or less sustained changes in both HR and PWA.

Significant correlations were observed during dSPT, aS, and aW between global movement magnitude and HR peaks (positive correlations, *r* = 0.38–0.46, *p* < 0.001), between global movement magnitude and PWA troughs (negative correlations, *r* = − 0.30 to −0.44, *p* < 0.001), and between HR peaks and PWA troughs (negative correlations, *r* = −0.41 to − 0.58, *p* < 0.001) (Supplementary Fig. 5). These results indicate that larger global movements were associated with greater cardiac sympathetic activation and/or parasympathetic withdrawal, as well as increased sympathetic vasoconstriction. At the individual level, these correlations were significant in 7 out of 9 subjects during dSPT, in 2 to 3 subjects during aS, and in 1 to 5 subjects during aW, depending on the specific variable pair considered.

## Discussion

We developed an automatic procedure for movement detection, with results closely matching those obtained by human observers manually scoring raw limb acceleration tracings. This approach provided a novel perspective on the physiological topography of nocturnal body movements and yielded findings consistent with expected differences between sleep and wakefulness and between the first and second parts of the night. Movement-related changes in HR were strikingly consistent with previous findings obtained using PSG, while simultaneous changes in PWA offered new insights into the underlying autonomic mechanisms.

We chose to present our primary results with reference to the dSPT, a practical approach relying on sleep diaries, which are recognized tools for collecting reliable sleep/wake data in field studies^24^. However, as dSPT may include wakefulness even in healthy subjects, we conducted a secondary analysis to score aS and aW based on actigraphy, which is recognized to have reasonable validity and reliability in healthy subjects^20^. This scoring was performed using the open-source algorithm proposed in 2015 by Van Hees et al.^25^, which processes raw wrist accelerometer data at high temporal resolution. The algorithm has been validated against PSG with good accuracy and its performance compares favorably with more recent machine-learning and deep-learning approaches^26^. Our finding of a substantially lower movement index during aS than during aW aligns with expectations and supports the internal consistency of our movement detection method. This reasoning is not circular: the Van Hees algorithm relies solely on changes in the orientation (angle) of the non-dominant arm, independent of the absolute acceleration of the limb or of movements of other body segments. Nevertheless, our use of sleep actigraphy to discriminate between aS and aW may help explain why the proportion of segmental movements involving the non-dominant wrist was higher during aW than during aS (39% vs. 26%, Fig. 1), although it should be noted that a similar trend was observed for the dominant wrist. Moreover, it is well established that the specificity of wrist actigraphy for detecting wakefulness is limited^27^, and that the Van Hees algorithm tends to overestimate the PSG-derived total sleep time^25^. As a result, aS is expected to include some periods of wakefulness, albeit limited. Despite this, our finding of a significantly different movement topography between aS and aW — specifically, a higher prevalence of segmental and lower-body movements during aS — suggests the presence of a distinct motor pattern during sleep. Furthermore, the observation that full-body, lower-body, and cross-regional movements were more frequent in the second half of the dSPT during aS, but not during aW, supports the hypothesis that many of these movements occurred during actual sleep. This is likely related to the increased prevalence of lighter (stage N1–N2) and REM sleep stages in the later part of the night.

Using only accelerometers and wearable electrocardiographic (ECG) sensors, we found that HR increases associated with movements began up to 6 s before movement onset and peaked approximately 3–5 s afterward. The striking similarity of this time course with results from analyses of LMS^10–14^ and LMMS^28^ recorded with PSG supports the validity of our movement detection method. This agreement is further strengthened by the knowledge that the electromechanical delay between the onset of electromyographic (EMG) activity, which is the signal recorded by PSG, and the onset of acceleration changes is negligible in this context^29^.

We report for the first time that nocturnal movements are consistently associated with distinct changes in PWA. Reductions in PWA measured at the finger are primarily attributable to sympathetic vasoconstriction of the digital vascular bed, mediated by α_1_-adrenergic receptors^23^. A similar interpretation is likely applicable to PWA measured at the wrist; however, previous studies^30,31^ have shown notable differences in PPG waveform characteristics between finger and wrist recordings. The regulation of the arm circulation is more complex, with weaker sympathetic vasoconstriction, greater β_2_-mediated sympathetic vasodilation, and lower PWA compared to the finger^23,32^. Therefore, PWA measurements at the arm or wrist should not be assumed to be equivalent to those obtained at the finger without direct quantitative validation, particularly in the context of nocturnal body movements.

In contrast, increases in HR may result from a variable combination of heightened sympathetic activity and reduced parasympathetic input to the heart^22^. Notably, the parasympathetic control of HR is faster than the sympathetic control of either HR^33^ or vascular resistance^34^. Thus, our observation that the onset and peak of the PWA decrease were temporally aligned with the onset and peak of the HR increase supports the hypothesis that sympathetic activation plays a prominent role in driving the changes in HR that precede the movement onset. This conclusion is supported by the significant correlation observed between the magnitude of the HR peak and that of the PWA trough, although the limited shared variance suggests that these metrics capture distinct, non-redundant aspects of autonomic regulation. While the procedure we used to synchronize the PPG and ECG time series did not account for the delay between the ECG R wave and the arrival of the arterial pulse at the wrist, this pulse arrival time — estimated at approximately 0.3 s at the finger^35^ — is negligible in the present context. Taken together, these findings raise the possibility that increased sympathetic activity may contribute to sleep fragmentation with motor events.

We also found evidence of a HR decrease below baseline, peaking approximately 10 s after the onset of segmental and regional, but not global, movements. Notably, the timing of this HR decrease closely corresponds with previous PSG-based reports on LMS^10,11,13,14^. While such post-movement HR reductions have also been described following periodic LMS accompanied by cortical arousal^11,12,28^, they may be more pronounced after periodic LMS without arousal^13,14^ and are altogether absent following short-interval aperiodic LMS doublets^11^ and LMMS with arousal^28^. Within the case of periodic LMS, this HR reduction has been observed to occur shortly after an increase in arterial blood pressure, which typically peaks 7–9 s after movement onset^13,14^. These observations support the hypothesis that the HR decrease below baseline reflects an arterial baroreflex response to the preceding blood pressure rise. This reflex, however, may be variably attenuated or overridden by central autonomic commands related to movement and/or arousal, depending on their intensity, consistent with predictions from mathematical models of human cardiovascular regulation^15^. The absence of a corresponding, time-aligned increase in PWA above baseline suggests a predominant parasympathetic contribution to the observed cardiac baroreflex response. Consistently, this HR decrease following periodic LMS has been reported to be more pronounced in school-age children than in adults^12^.

Our analysis of global movements during aS revealed a previously undescribed pattern: a late increase in PWA above baseline, peaking 20–30 s after movement onset, accompanied by a corresponding late decrease in HR, with a trough occurring around 30 s after onset. This pattern may reflect delayed changes in sympathetic outflow to the heart and vasculature, potentially mediated by baroreflex activation in response to a preceding rise in arterial blood pressure, or by the resolution of arousal responses or brief awakenings associated with the movements.

We conducted a quantitative analysis of the relationships between movement magnitude, HR peak, and PWA trough at both the group and individual levels, with the dual aim of assessing the internal consistency of our results and evaluating the potential for developing digital biomarkers based on movement-related cardiovascular changes during sleep. Such biomarkers could be derived non-obtrusively using a fully wearable sensor array, without requiring subjects to perform standardized motor tasks during the day. If these biomarkers were found to be associated with cardiovascular risk, they could offer substantial clinical utility. Correlations between the movement magnitude and either the HR peak or the PWA trough were negligible following segmental movements, suggesting that the cardiovascular responses to these movements are relatively stereotyped. Evidence of this correlation emerged primarily in association with movements of greater complexity, particularly global movements during dSPT, where it reached statistical significance in most subjects. This may be partly attributable to the higher frequency of such movements compared to those occurring during aS or aW. Ratios between the HR peak or PWA trough and movement magnitude during dSPT thus represent promising candidates for cardiovascular biomarkers to be explored in future studies. In parallel, the movement index and topography metrics may provide useful indicators of sleep fragmentation or central motor disorders, while purely autonomic metrics derived from HR and PWA could offer integrated information on sleep quality and autonomic dysfunction.

An important limitation of this study is the absence of gold-standard PSG validation, which restricts direct comparisons with established markers of sleep and arousal, including PSG-defined sleep stages and cortical arousals. This is particularly relevant given our reliance on actigraphy for sleep-wake classification, which is known to overestimate sleep and may misclassify periods of quiet wakefulness, as discussed above. Additionally, our analysis was restricted to artifact-free, isolated movements separated by at least 30 s, thereby excluding more complex motor patterns involving clustered or overlapping events. The absence of continuous blood pressure monitoring further limits our ability to confirm the hypothesized baroreflex mechanisms underlying delayed HR and PWA changes.

We also acknowledge that our choice to detect and quantify movement magnitude based on the envelope of the acceleration signal vector magnitude was arbitrary, as was our method of estimating the magnitude of regional and global movements by summing the maximum differences in the envelope of the acceleration signal vector magnitude across individual body segments. While intuitively related to movement amplitude, these metrics do not fully capture the biomechanical complexity of movement dynamics (i.e., body segment mass, joint angular acceleration, and neuromuscular control). Nonetheless, our movement detection procedure reliably reproduced the results of visual inspection of raw accelerometric tracings and showed significant correlations with peak changes in HR and PWA, particularly during global movements. Future studies involving larger samples of healthy individuals and clinical populations with sleep and/or motor disorders across different age groups (including children and older adults) will be essential to further refine and validate this approach, especially through comparison with simultaneous video-PSG recordings. Moreover, future refinements should explore the potential informational value of angular velocity data from gyroscopes in the wearable inertial measurement units, which was not examined in this initial proof-of-concept study. Finally, and importantly, our current data do not allow us to draw conclusions regarding the sensitivity or precision of our manual annotation of accelerometer tracings for identifying movements observed in video-PSG. Nevertheless, differences in results can be expected on physiological and biomechanical grounds, depending on whether motor events during sleep are scored using EMG signals, accelerometer data, or video recordings. By definition, movements are characterized by the presence of linear acceleration or angular velocity of a body segment. Subtle EMG activations may not translate into actual segmental motion, whereas small movements occurring during sleep may remain undetected in video analysis, because of camera resolution limits, restricted field of view, partial obstruction by bed covers, low contrast between the subject and background, or insufficient frame rate to capture brief or low-amplitude displacements. Therefore, the correspondence between motor events identified through EMG, acceleration and angular velocity measures, and video analysis should be the focus of systematic investigation.

In conclusion, this study provides proof-of-concept evidence for the feasibility and internal consistency of a novel approach to detecting and quantifying body movements and their cardiovascular correlates during nocturnal sleep and wakefulness using a wearable sensor array. This method allowed us to characterize the physiological landscape of these movements and their associated cardiovascular responses, laying the groundwork for the future development of digital, scalable nighttime biomarkers of cardiovascular and motor function at the population level.

## Methods

### Dataset

The study was conducted on the LOOKING-GLASS MIAR (Integrated Rest-Activity Monitoring), dataset, which included 12 healthy volunteers (6 females; age 30.7 ± 1.7 years; body weight 69.4 ± 2.1 kg; 11 right-handed and 1 ambidextrous) recruited at the University of Bologna, Italy. All subjects signed an informed consent form prior to participation. The study protocol was reviewed and approved by the Ethics Committee of the University of Bologna (protocol number 0290278) and was conducted in accordance with the principles outlined in the Declaration of Helsinki.

The subjects wore five inertial measurement units (AX6, Axivity, UK) measuring triaxial acceleration at 100 Hz at the level of both wrists, both ankles, and the lower back (L5, right paramedian position). A four-channel armband photoplethysmograph (PPG; Verity Sense, Polar, Finland) was used to record data at a sampling rate of 55 Hz, while a single-lead wearable chest-belt ECG (H10, Polar, Finland) was recorded at 130 Hz. Sensor synchronization was achieved mechanically by gently tapping each device, in a predefined sequence, against an external reference AX6 sensor. This procedure generated distinct bursts of acceleration detectable by all devices, and the abrupt onset of these signals was used as a temporal marker for alignment. The method leverages the built-in triaxial accelerometers present in both the Verity Sense and H10 sensors. Detailed information about the synchronization procedure is provided in the Supplementary Materials, including Supplementary Figs. 6 and 7. The accelerometer data recorded by the ECG and PPG sensors were not utilized for any other analyses in the present study. The raw data measured by the Polar H10 and Verity Sense devices were acquired using a customized mobile application (VEGA) developed in our laboratory for this purpose, which operates via Bluetooth on a smartphone. In three participants (two females), the ECG and PPG data were missing due to technical issues with the data connection from the H10 and Verity Sense devices to the smartphone. The participants wore all wearable sensors for 23.1 ± 0.3 h (mean ± SEM), including a full night’s sleep at their premises, and compiled a sleep diary.

### Sleep assessment

Sleep and wakefulness during dSPT were automatically scored using the non-dominant wrist accelerometer and the algorithm validated by Van Hees et al.^25^ against PSG and implemented in the open-source GGIR software package^36^. Unlike traditional actigraphy methods based on device-specific activity counts, this algorithm uses raw triaxial acceleration to identify nocturnal sleep as sustained bouts of inactivity lasting 5 min or longer. Inactivity is defined as the absence of changes in arm angle greater than 5°, estimated from triaxial acceleration data within the dSPT. We used the Van Hees algorithm^25^ to classify each 5-s epoch as either aS, corresponding to sustained inactivity, or aW. From the resulting time series, we extracted the total duration of aS and aW, sleep efficiency (i.e., the percentage of dSPT spent in aS), and the number of actigraphy-defined awakenings, defined as episodes of aW occurring between periods of sustained inactivity, normalized per hour of aS.

### Movement detection procedure

We developed a threshold-based procedure to detect movements of each of the five body segments equipped with wearable accelerometers (i.e., both ankles, both wrists, and the lower back) during dSPT. The procedure begins by computing the signal vector magnitude of the raw triaxial acceleration data (ACC_SVM_), defined as the Euclidean norm of the three acceleration components:

$$\:{ACC}_{SVM}=\:\sqrt{{acc}_{x}^{2}+{acc}_{y}^{2}\:+{acc}_{z}^{2}}$$.

The upper and lower envelopes of the band-pass filtered (0.1–10 Hz) ACC_SVM_ are then extracted by identifying local minima and maxima⁠⁠. Specifically, local minima are defined as points where the first derivative of the ACC_SVM_ changes sign from negative to positive, and local maxima as points where the sign changes from positive to negative. These extrema are grouped into larger segments, and global maxima within each segment are identified to construct a smoother, noise-robust envelope. The refined extrema are subsequently interpolated to generate the final upper and lower envelopes of ACC_SVM_. Movements are then detected using a threshold-crossing algorithm based on the difference between the two envelopes (see details below). The entire procedure is illustrated in Supplementary Fig. 8.

### Optimization and validation of movement detection thresholds

The movement detection thresholds were optimized and validated against visual annotations of raw ACC_SVM_ data. Two independent raters (biomedical engineers with experience in signal processing, MS and PDF) manually annotated movement onset and offset times using raw ACC_SVM_ signals from 6 h of recordings per subject: 2 h each from a wrist, an ankle, and the lower back sensor. The three time windows were non-overlapping to ensure a broad sampling of movement types across different body segments. The inter-rater agreement was calculated as the ratio of overlapping annotations to the total number of annotations made by both raters. Discrepancies were resolved through consensus to produce the final annotated dataset.

The movement detection procedure was performed over the same 6-hour period with 15 different thresholds for each body part (Supplementary Fig. 1). The output of the procedure consisted of the onset and offset times of each detected movement burst. These were compared with the manual annotations to compute the following metrics: true positives (TP, the number of automatically detected movements that overlapped at least partially with a visually annotated movement), false positives (FP, the number of automatically detected movements with no overlap with a visually annotated movement), and false negatives (FN, the number of visually annotated movements with no corresponding automatic detection). The sensitivity (TP/(TP + FN)), precision (TP/(TP + FP)), and F1-score (TP/(TP + (FP + FN)/2)) were then computed. The optimal detection threshold for movements of each body segment was selected based on the maximum mean F1-score across subjects and used in all subsequent analyses.

### Movement definition and characterization

The movement detection procedure was independently applied to each body segment using the corresponding optimized detection threshold determined in the previous section. For each detected movement, the procedure yielded the start and end times as well as the maximum difference between the upper and lower ACC_SVM_ envelopes (Fig. 1 panel c). This maximum envelope difference was used to quantify movement magnitude, reflecting the peak accelerations of each body segment.

Movements that overlapped in time across different body segments were treated as a single composite movement, with magnitude quantified as the sum of the maximum ACC_SVM_ envelope differences from each contributing segment. For example, if a movement started with the ankle and overlapped with another movement involving the lower back, and the lower back movement continued after the ankle movement ended, the composite movement magnitude would be the sum of the maximum envelope differences from both the ankle and the lower back segments. Movement duration was defined as the time between the onset (upward threshold crossing of the first sensor involved) and the offset (downward threshold crossing of the last sensor involved). Two movements were considered distinct if separated by an interval of at least 0.5 s from the offset of one movement to the onset of the next movement^6^. This approach enabled the characterization of 31 possible combinations of movements across the five body segments equipped with accelerometers and the analysis of their distribution throughout the dSPT.

Movements were classified as segmental (or localized) when confined to a single body segment^1^, and as multisegmental when involving two or more segments.

Multisegmental movements were further categorized into the following subtypes:

- Global movements: involving both wrists, both ankles, and the lower back, indicative of whole-body activation.
- Regional movements, including:
  - Upper-body movements: involving both wrists, or one wrist and the lower back.
  - Lower-body movements: involving both ankles, or one ankle and the lower back.
  - Cross-regional movements: comprising any other combination of non-contiguous segments.

Postural changes were identified as global movements when accompanied by shifts of at least 30° in the orientation of the lower back accelerometer^37^. In addition, upright posture was detected when the gravitational force along the vertical axis fell below − 0.66 *g*^27^; such instances likely reflected unreported episodes of sitting or walking during the dSPT and were excluded from further analysis.

For each detected movement, the duration and the maximum difference in the ACC_SVM_ envelope were extracted. The movement index, defined as the number of movements per unit time, was also calculated for each subject and movement category. All features were aggregated by subject and movement type. These analyses were conducted across the entire dSPT as well as within aS and aW, either globally or separately for the first and second halves of the dSPT.

### Heart rate and pulse wave amplitude analysis

The peaks of the R waves in the ECG were detected using the Neurokit algorithm^38^, which was recently benchmarked as one of the top R-wave peak detectors from single-lead ECG^39^. The systolic peaks and diastolic feet of the PPG were detected using the Aboy + + algorithm implemented in pyPPG^40^, which was recently benchmarked as one of the top PPG beat detectors^41^. The PWA was defined as the difference between the PPG peak and PPG foot within the same cardiac cycle.

**Fig. 5:**
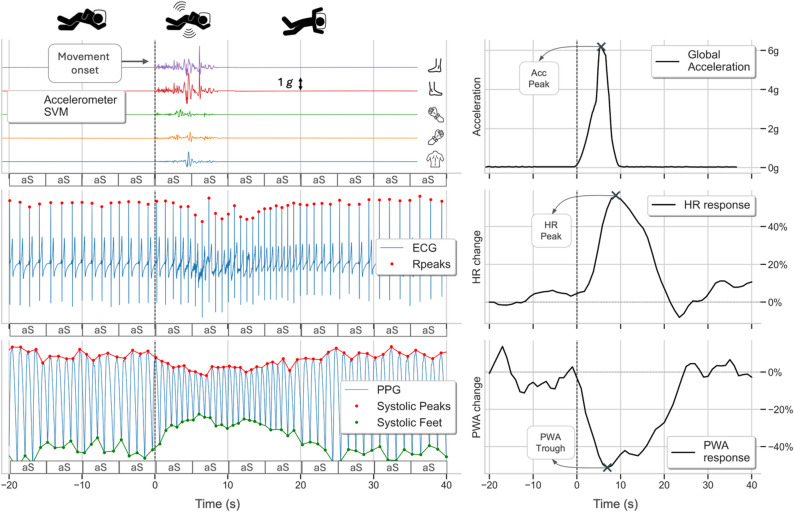
Processing of the acceleration signal vector magnitude (SVM, top), electrocardiogram (ECG, middle), and photoplethysmogram (PPG, bottom) for a representative global movement occurring during actigraphy-defined sleep. The panels on the right display the envelope difference of the acceleration SVM (top), along with the corresponding heart rate (HR, middle) and pulse wave amplitude (PWA, bottom) responses.

Beat-to-beat changes in HR and PWA associated with movements were extracted within fixed time windows spanning from 19 s before to 40 s after the onset of each detected movement. The data were linearly interpolated at 1 Hz. Following the approach used in a previous study^12^, we included only movements that were isolated, i.e., not preceded nor followed by another movement within a 30-s window. Motion artifacts affecting the HR and PPG time series were manually annotated using a custom graphical user interface. Segments shorter than 10 s were corrected via cubic spline interpolation, while longer segments were discarded. The average HR or PWA value over the first 10 s of each movement-related time window (i.e., from − 19 s to − 9 s before movement onset) was used as the baseline and subtracted from each value within the window^11^. The resulting difference was then divided by the baseline value to obtain normalized HR and PWA time series, expressed as a percentage relative to the pre-movement baseline, which was set at 0%. From these normalized time series, the HR peak – defined as the maximum increase in HR above baseline following movement onset - and the PWA trough – defined as the maximum decrease in PWA below baseline after movement onset - were computed (Fig. 5).

### Statistical analyses

The normality of data distributions was assessed using the Shapiro-Wilk test. Depending on the outcome, either a paired t-test (for normally distributed data) or a Wilcoxon signed-rank test (for non-normally distributed data) was applied for pairwise comparisons. Statistical significance was defined as *p* < 0.05. Results are reported as mean ± standard error of the mean (SEM), with N indicating the number of subjects.

The time courses of cardiac activation were first averaged within individuals and then across individuals to obtain grand averages. Second-by-second one-sample t-tests vs. 0% were performed to assess changes in HR and PWA relative to the pre-movement baseline. Additional comparisons were made to evaluate differences in HR and PWA responses across various movement types: wrist vs. ankle segmental movements, upper-body, lower-body, and cross-regional movements, as well as global movements.

To further investigate the relationship between movement intensity and cardiovascular responses, we fitted three linear mixed-effects models: (1) HR peak as a function of movement magnitude (defined as the maximum difference in ACC_SVM_ envelope), (2) PWA trough as a function of movement magnitude, and (3) PWA trough as a function of HR peak. In each model, the dependent variable was regressed on the fixed-effect predictor, with subject-specific random intercepts included to account for inter-subject variability. Model coefficients were tested for statistical significance using z-tests, with *p* < 0.05 considered significant. All analyses were performed in Python using the statsmodels library.

## Supplementary Information

Below is the link to the electronic supplementary material.

Supplementary Material 1

## Data Availability

The underlying code for this study is publicly available at https://github.com/marcellosicbaldi/Every-Move-You-Make.
