# Supplementary material for: Mapping the physiological landscape of body movements during nocturnal sleep and wakefulness and their cardiovascular correlates with a wearable multi-sensor array
Source: Sci Rep. 2025 Nov 29;16:296. doi: 10.1038/s41598-025-29723-7 (PMC12770513; doi:10.1038/s41598-025-29723-7)
Supplement: Supplementary file 1 — Supplementary Material 1 [file 41598_2025_29723_MOESM1_ESM.pdf]

Mapping the physiological landscape of body movements during nocturnal sleep and wakefulness and their cardiovascular correlate with a wearable multi-sensor array

Supplementary Methods

Sensor synchronization

Recording start

At the start of the recording, all AX6 devices were synchronized by initializing them simultaneously using the Axivity software on the same PC. The Polar H10 and Verity Sense devices lacked a built-in synchronization mechanism, meaning their internal clocks could exhibit slight time shifts relative to each other and AX6 sensors. To address this, a mechanical synchronization procedure was performed. Specifically, a reference AX6 device was bumped three times against the Polar H10, then three times against the Polar Verity Sense, and finally, the H10 and Verity Sense were bumped against each other three times. After this procedure, all three sensors were held into an evaluator's hand, who performed five shoulder rotations around the medio-lateral axis. The accelerometer peaks generated by the mechanical bumps were used to determine the time shift of the Polar H10 and Verity Sense relative to the reference AX6. These offsets were then applied to adjust the timestamps of the Polar H10 and Verity Sense. The shoulder rotations served as a visual validation of the synchronization (Supplementary Figure 1).

Polar devices disconnections

Polar devices streamed data via Bluetooth to a smartphone using an app (VEGA) developed in the laboratory. If the Bluetooth connection was lost (e.g., due to the subject moving too far from the phone), data gaps occurred. Once the subject moved back within range, the sensors automatically reconnected. However, this reconnection caused the timestamps to reset, disrupting the synchronization. The re-synchronization was tackled differently for the H10 and Verity Sense devices as explained hereafter.

Re-synchronization of H10 devices

Three participants experienced a disconnection of the H10 device before the sleep period. For these participants, the H10 device was re-synchronized using the AX6 device placed on the lower back. This was achieved by visually identifying walking bouts after the last disconnection before sleep. Walking produces distinct acceleration peaks in both the chest (H10) and lumbar (AX6) accelerometer signal magnitude vectors. These peaks were used to determine the time shift between

the H10 and the lumbar AX6 devices, following the same approach as for mechanical bumps. After this procedure, the Polar H10 and all AX6 devices were re-synchronized for the sleep period.

Re-synchronization of Verity Sense devices

The Polar Verity Sense device was re-synchronized with the Polar H10 device (and, therefore, with the AX6 devices) during the sleep period time by identifying the time lag that maximized the cross-correlation function between RR interval and pulse-to-pulse (PP) interval time-series during artifact-free segments (Supplementary Figure 2). This procedure was performed only once per subject, as no disconnections occurred during the sleep period for any subject.

Supplementary Table 1.

	Index (n/h)			Duration (s)			Magnitude (mg)		
	dSPT	aS	aW	dSPT	aS	aW	dSPT	aS	aW
SEG	9.4 ± 0.3	9.0 ± 0.3	14.7 ± 0.6	1.4 ± 0.0	1.4 ± 0.0	1.8 ± 0.1	36 ± 1	36 ± 1	41 ± 1
NDA	2.3 ± 0.1	2.0 ± 0.1	3.3 ± 0.3	1.5 ± 0.0	1.5 ± 0.0	1.3 ± 0.1	30 ± 1	30 ± 1	29 ± 1
DA	2.4 ± 0.1	2.3 ± 0.1	2.3 ± 0.2	1.5 ± 0.0	1.6 ± 0.0	1.2 ± 0.1	29 ± 1	29 ± 1	33 ± 2
NDW	2.9 ± 0.1	2.3 ± 0.1	6.7 ± 0.5	1.3 ± 0.0	1.2 ± 0.0	1.4 ± 0.0	40 ± 1	38 ± 1	37 ± 1
DW	2.8 ± 0.1	2.3 ± 0.1	4.6 ± 0.2	1.7 ± 0.1	1.5 ± 0.0	2.3 ± 0.2	47 ± 1	47 ± 2	55 ± 4
T	0.9 ± 0.1	0.9 ± 0.1	1.8 ± 0.1	1.2 ± 0.1	1.3 ± 0.1	0.9 ± 0.1	21 ± 0.3	21 ± 0.3	19 ± 0.3
REG	8.0 ± 0.2	7.1 ± 0.2	17.6 ± 0.7	4.4 ± 0.1	4.2 ± 0.1	5.4 ± 0.1	224 ± 9	184 ± 6	386 ± 20
LB	3.4 ± 0.2	3.2 ± 0.2	6.2 ± 0.3	3.5 ± 0.1	3.5 ± 0.1	3.8 ± 0.2	108 ± 4	106 ± 3	159 ± 19
UB	1.1 ± 0.1	1.0 ± 0.1	3.6 ± 0.1	4.7 ± 0.3	3.6 ± 0.1	8.3 ± 0.7	247 ± 15	203 ± 16	376 ± 17
CR	3.5 ± 0.1	3.0 ± 0.1	9.9 ± 0.6	5.3 ± 0.1	5.5 ± 0.2	5.5 ± 0.1	311 ± 12	258 ± 7	475 ± 22
GLOB	10.3 ± 0.4	5.2 ± 0.3	59.5 ± 1.3	17.4 ± 0.6	10.3 ± 0.3	23.8 ± 0.8	2471 ± 62	1163 ± 38	3628 ± 80

Movement index, duration, and magnitude (cumulative peak differences between the envelopes of the acceleration signal magnitude vector) are reported during diary-defined sleep period time (dSPT), and actigraphy-defined sleep (aS) and wakefulness (aW). SEG, segmental movements. NDA and DA, non-dominant and dominant ankle movements, respectively. NDW and DW, non-dominant and dominant wrist movements, respectively. T, trunk movements. REG, regional movements. LB and UB, lower- and upper-body movements, respectively. CR, cross-regional movements. GLOB, global movements. Values are reported as mean ± SEM, with N = 12 subjects.

Supplementary Figure 1. Sensitivity, precision, and F1-score for automatic movement detection as a function of detection thresholds.

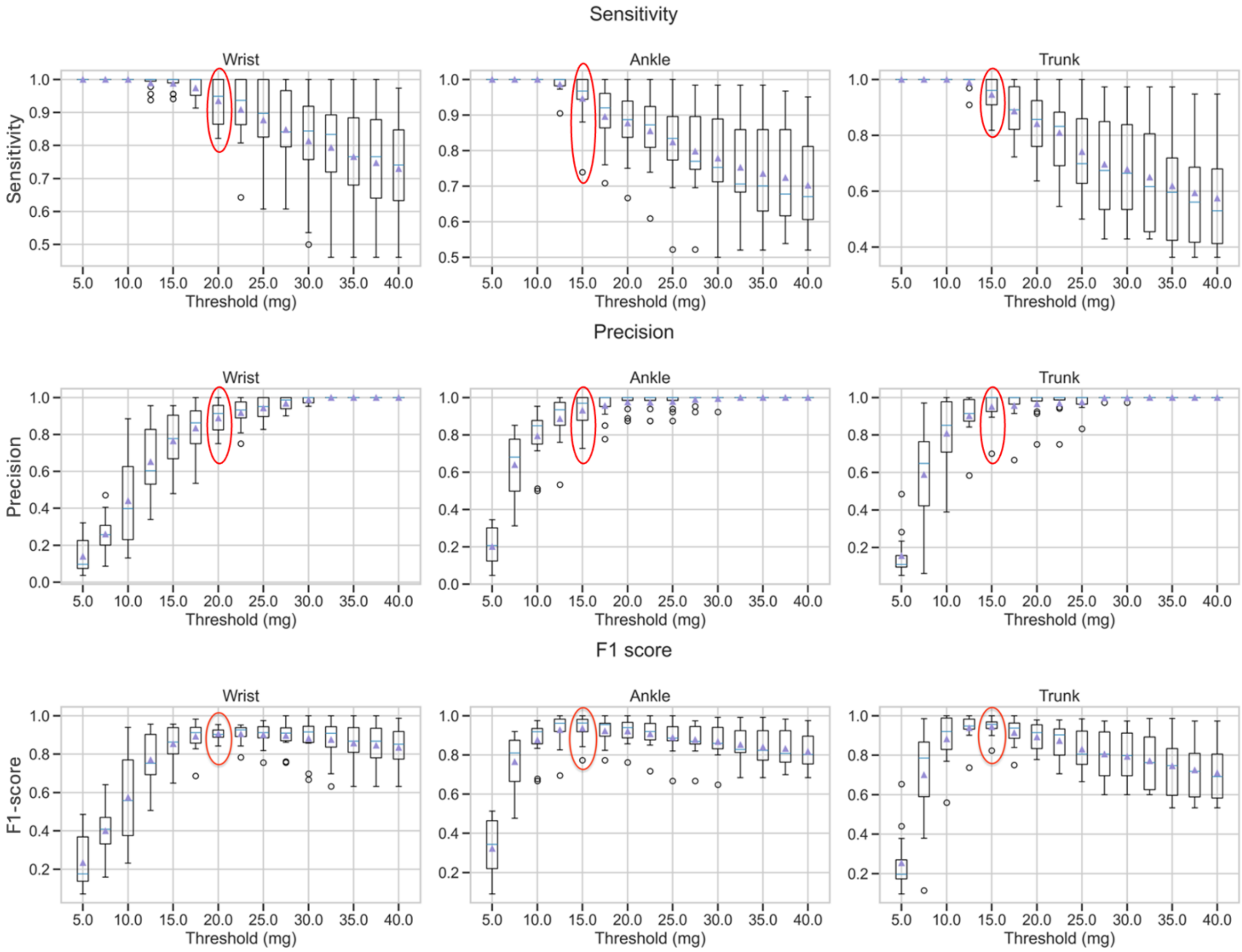

Ovals indicate the thresholds selected based on F1-scores. The values of sensitivity, precision, and F1-score at the selected thresholds for the wrist were 93.5 ± 6.7%, 88.8 ± 7.9%, and 90.6 ± 3.1%, respectively. The corresponding values for the ankles were 94.7 ± 7.2%, 93.2 ± 8.7%, and 93.7 ± 6.7%, respectively, and those for the trunk were 94.6 ± 5.9%, 95.1 ± 8.4%, and 94.4 ± 4.5%, respectively. Data are shown here as boxplots with N = 12 subjects.

Supplementary Figure 2. Movement occurrence rate across the diary-defined sleep period.

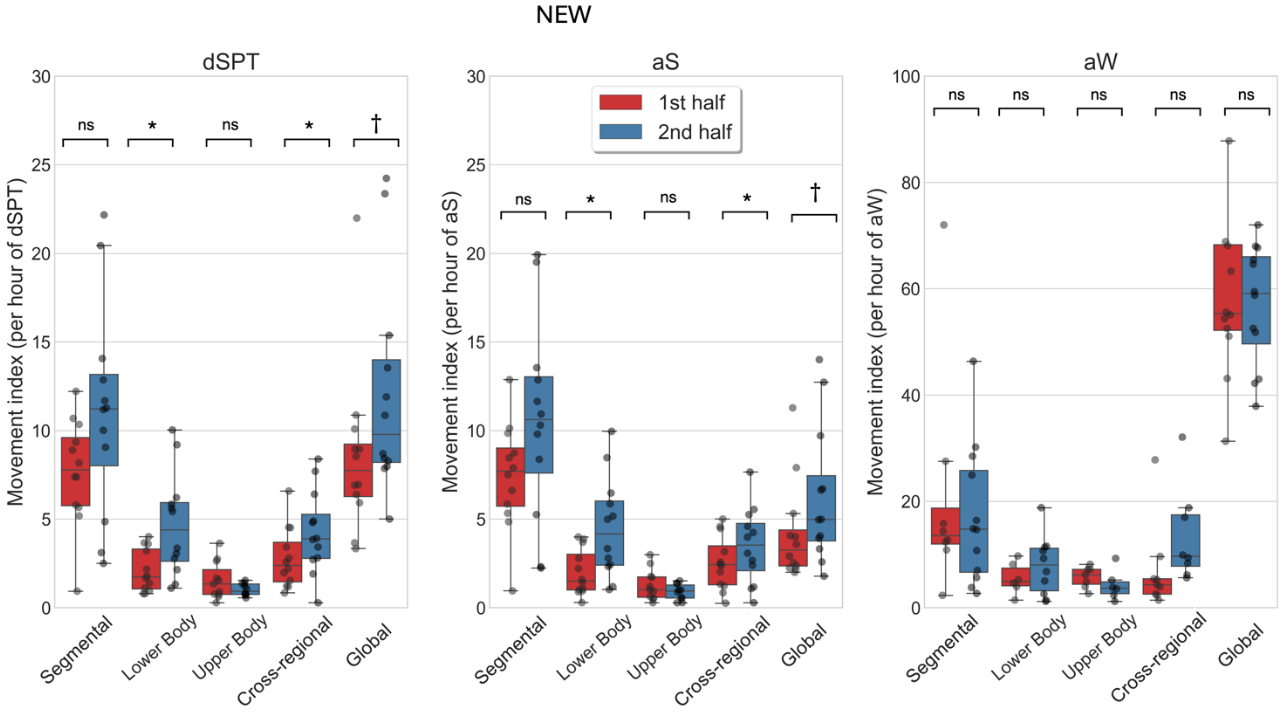

Movement index during the first and second half of the diary-defined sleep period time (dSPT) and of actigraphy-defined sleep (aS) and wakefulness (aW) for each movement category. Points represents individual subject results (N = 12). *: $p < 0.05$, paired t-test; †: $p < 0.05$, Wilcoxon test; ns: not significant.

Supplementary Figure 3. Relationships between movement magnitude, heart rate peak, and pulse wave amplitude trough for segmental wrist and ankle movements during the diary-defined sleep period.

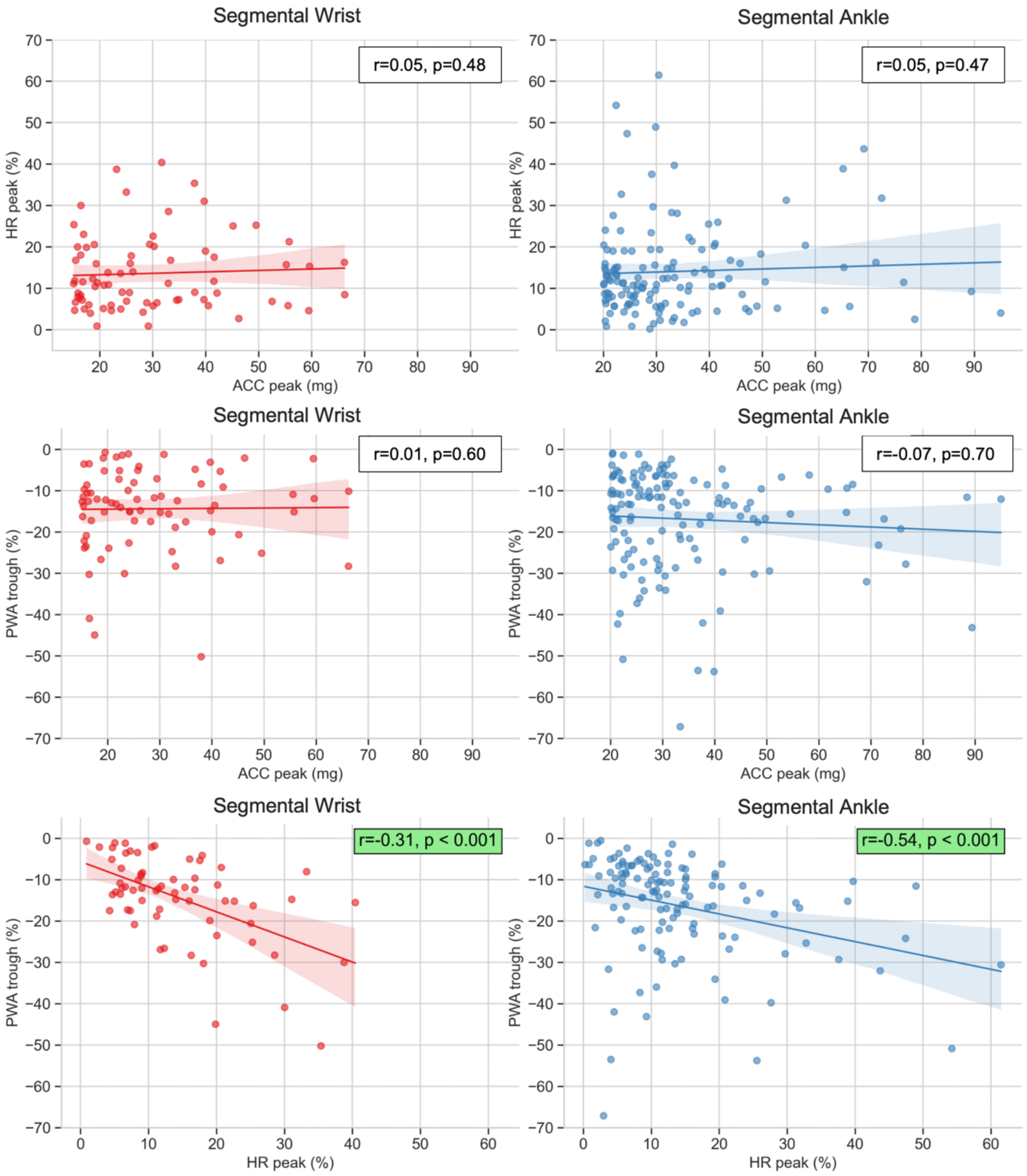

ACC peak, peak-to-peak amplitude of the acceleration signal magnitude vector. HR, heart rate. PWA, pulse wave amplitude. Shaded areas represent 95% confidence intervals; r is the Pearson's correlation coefficient, and p values are based on linear mixed effect models. The green legend indicates a significant correlation. Points indicate individual movements.

Supplementary Figure 4. Relationships between movement magnitude, heart rate peak, and pulse wave amplitude trough for regional movements during the diary-defined sleep period.

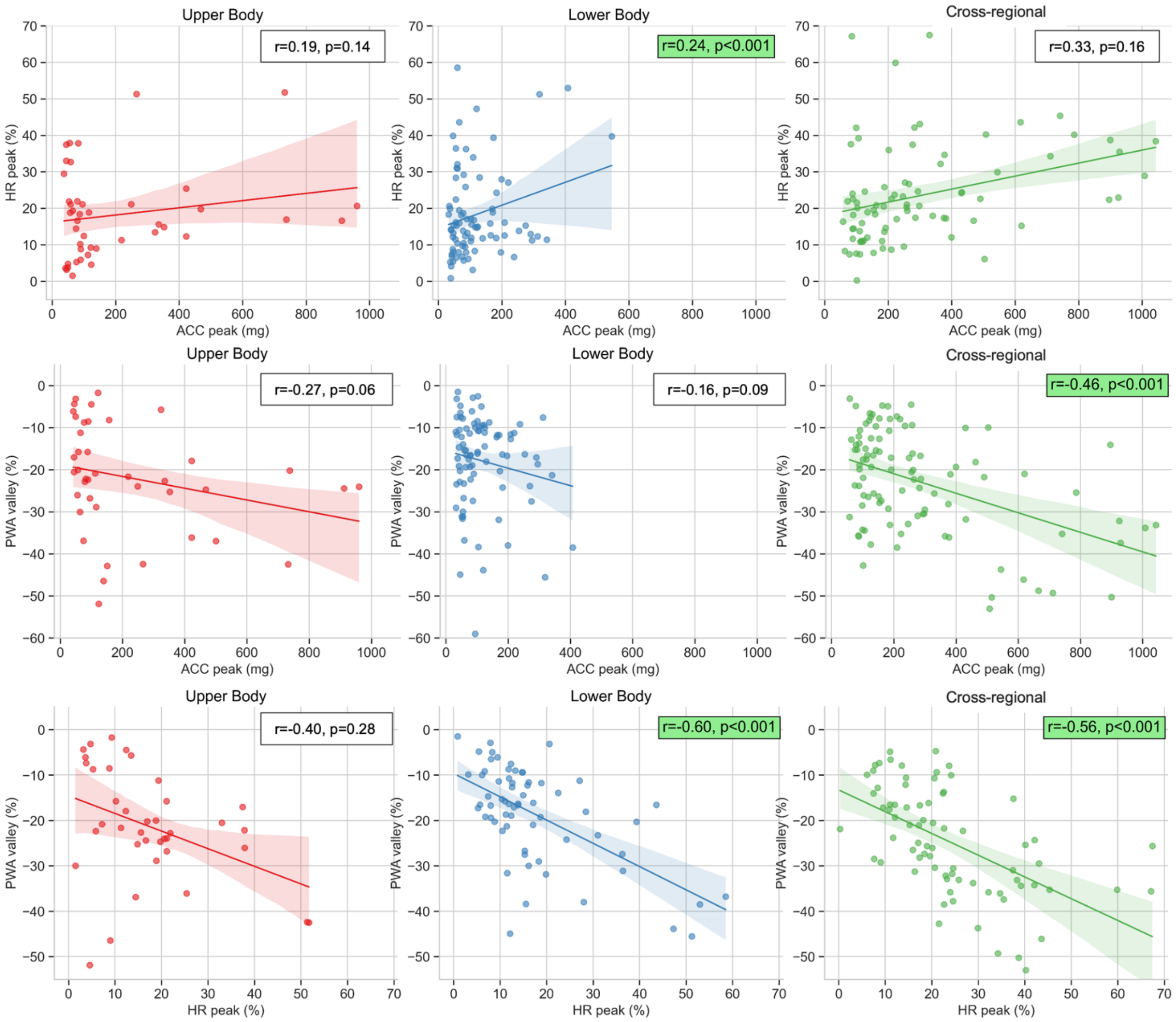

ACC peak, peak-to-peak amplitude of the acceleration signal magnitude vector. HR, heart rate. PWA, pulse wave amplitude. Shaded areas represent 95% confidence intervals; r is the Pearson's correlation coefficient, and p values are based on linear mixed effect models. The green legend indicates a significant correlation. Points indicate individual movements.

Supplementary Figure 5. Relationships between movement magnitude, heart rate peak, and pulse wave amplitude trough for global movements during the diary-defined sleep period.

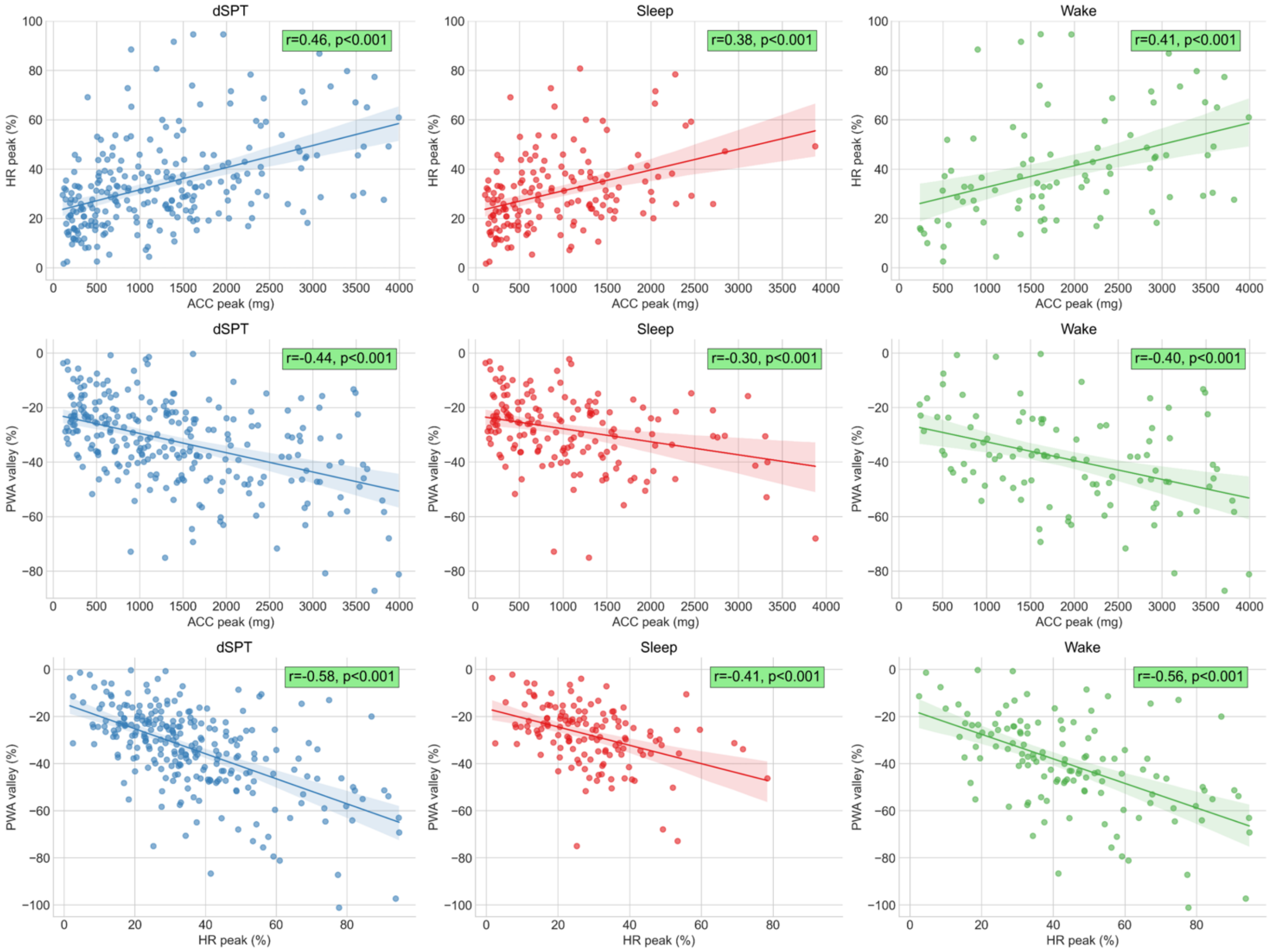

ACC peak, peak-to-peak amplitude of the acceleration signal magnitude vector. HR, heart rate. PWA, pulse wave amplitude. dSPT, diary-derived sleep period time. aS and aW, actigraphy-derived sleep and wakefulness, respectively. Shaded areas represent 95% confidence intervals; r is the Pearson's correlation coefficient, and p values are based on linear mixed effect models. The green legend indicates a significant correlation. Points indicate individual movements.

Supplementary Figure 6. Synchronization between all devices at the start of the recording.

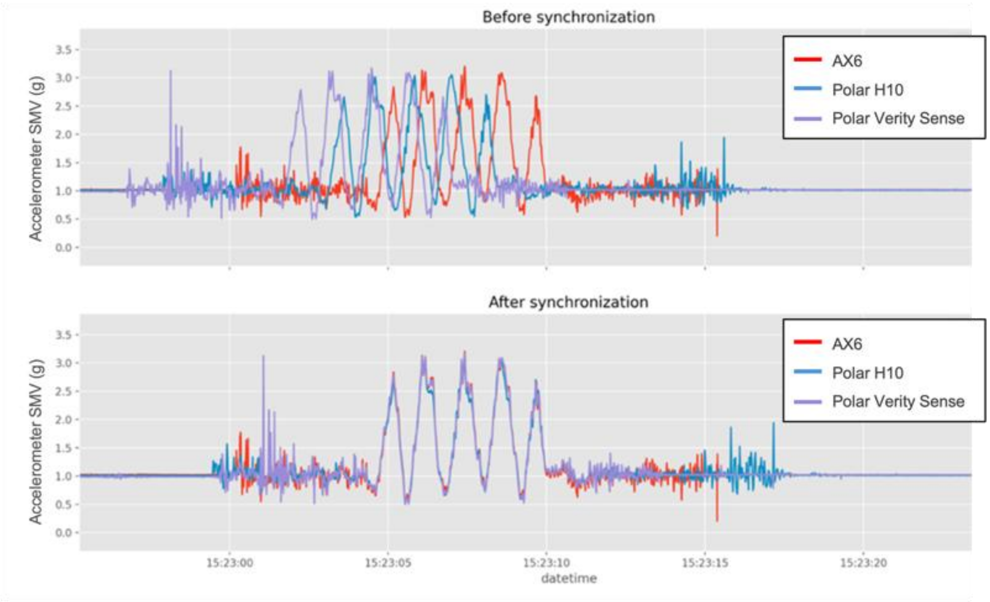

Representative example of the results of the mechanical synchronization of the Axivity AX6, Polar H10, and Polar Verity Sense wearable devices.

Supplementary figure 7. Synchronization between Polar devices.

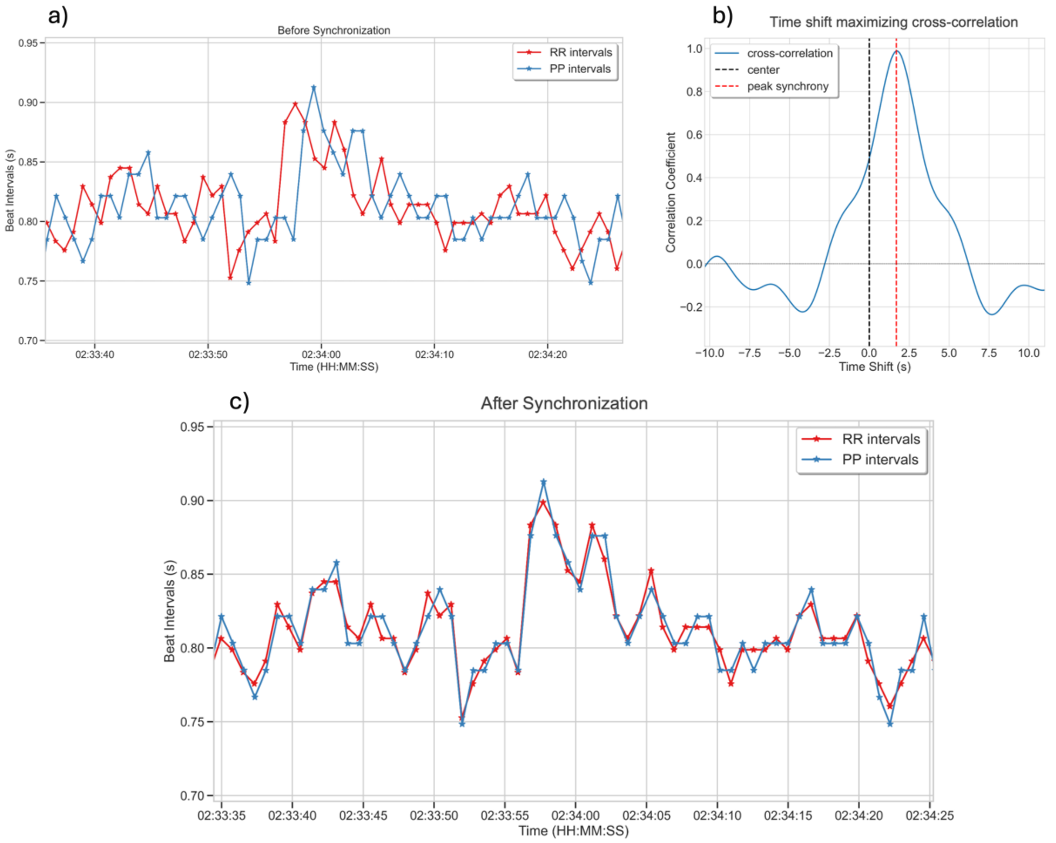

Representative example of the synchronization between pulse-to-pulse (PP) intervals extracted from the Polar Verity Sense wearable photoplethysmography device and RR intervals extracted from the Polar H10 wearable electrocardiographic device. Due to sensor Bluetooth disconnections, there was an offset of 1.7 s between the two signals (panel a), This offset was found as the time shift maximizing the cross-correlation function between the RR and PP interval time-series (panel b), and was then applied to the PP interval time-series to achieve synchronization (panel c).

Supplementary Figure 8. Movement detection from accelerometer data.

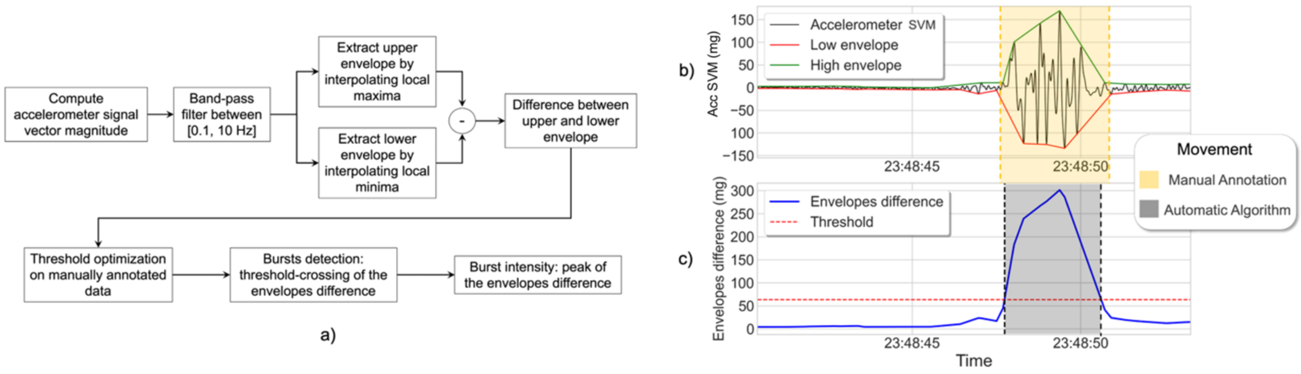

Panel a illustrates the steps of the algorithm. Panel b shows the raw accelerometer signal vector magnitude (Acc SVM) of the left wrist accelerometer signals (black) and its upper (green) and lower (red) envelopes. The corresponding difference between the upper and lower envelopes is shown in panel c. The movement onset is defined as the time point where the Acc SVM envelope difference crosses a predefined threshold.
